# COVID‐19 Vaccination in Canadian Dental Schools

**DOI:** 10.1111/jphd.12670

**Published:** 2025-03-21

**Authors:** Isabella Turquete, Sreenath Madathil, Paul J. Allison

**Affiliations:** ^1^ Faculty of Dental Medicine and Oral Health Sciences McGill University Montreal Quebec Canada

**Keywords:** COVID‐19 vaccine, dental students, healthcare workers vaccination, vaccine acceptance, vaccine hesitancy

## Abstract

**Background:**

Oral Healthcare workers, including dental students, face a great risk of COVID‐19 infection. High COVID‐19 vaccination coverage is essential for a protected workforce. This study, which aims to document the COVID‐19 vaccination experience among dental students and employees from Canadian dental schools during the COVID‐19 pandemic, provides crucial insights that can significantly impact future vaccination strategies.

**Methods:**

This study used data from a prospective cohort conducted between April 2021 and May 2022. We recruited 600 participants, including dental students, faculty, and support staff from 10 Canadian dental schools. Data were collected monthly from all subjects. Vaccination acceptance and vaccination time were assessed. Logistic regression models were performed to identify predictors of COVID‐19 vaccine acceptance and late vaccination. In order to detect hesitation tendencies, descriptive statistics were used to observe the distribution of time to vaccination between age groups of employees and students.

**Results:**

Out of 600 participants at baseline (70% female; average age 36 years old), 91% received at least one dose of the COVID‐19 vaccine. No associations were found between sociodemographic factors and COVID‐19 vaccine acceptance. Individuals aged 50–59 were less likely to delay the vaccination than most of our sample. Students presented more outliers for later vaccination times, particularly in younger age groups.

**Conclusion:**

High vaccination acceptance among dental students is crucial for promoting professionalism and influencing patients. Integrating vaccine advocacy into their education might enhance vaccination uptake in the general population.

## 1 Introduction

Oral healthcare settings pose a cross‐infection risk between professionals and patients due to their proximity and aerosol‐generating procedures [1]. Thus, Oral Health Professionals (OHP) are among the most vulnerable healthcare workers (HCWs) to COVID‐19 infection [2]. Studies have reported the prevalence of SARS‐CoV‐2 among oral healthcare professionals in different countries [3, 4, 5, 6].

Since COVID‐19 vaccines were distributed, they have effectively decreased disease severity [7]. In December 2020, Health Canada authorized the first COVID‐19 vaccine, the Pfizer‐BioNTech. The priority groups to receive the vaccine were the HCWs, elderly long‐term care residents, and adults residing in remote and isolated Indigenous communities [8]. The Canadian Dental Association (CDA) then encouraged OHP to get the COVID‐19 vaccine as soon as it became available [9]. Vaccination was not mandatory in Canada but was strongly recommended by provincial and university authorities. To increase vaccination rates, local and national public health authorities implemented proof‐of‐vaccination requirements or certificates, aiming to reduce viral transmission and incentivize vaccination. By September 2021, 10 Canadian provinces (Quebec, British Columbia, Manitoba, Ontario, Nova Scotia, Alberta, New Brunswick, Saskatchewan, Newfoundland, and Prince Edward Island) had established vaccine programs. The timeline for implementing these programs varied across provinces [10].

Despite priority access to COVID‐19 immunization, general HCWs and dental professionals demonstrated concerns and certain hesitancy towards COVID‐19 vaccines [11, 12, 13, 14, 15, 16, 17]. Vaccine hesitancy is a “delay in accepting or refusing vaccines despite availability of vaccination services” [18, 19]. In 2019, the World Health Organization (WHO) identified vaccine hesitancy as one of the top 10 global health threats [20]. Healthcare professionals play a key role in combating vaccine hesitancy as they are trusted sources of advice [21].

Vaccine hesitancy has been reported among dental students in different parts of the world [13, 14, 15]. However, more data is needed for this group in Canada, highlighting the necessity for further research. In this context, this study aims to document the COVID‐19 vaccination experience among students, faculty, and support staff in Canadian dental schools during the COVID‐19 pandemic and to observe the trends in vaccination time, comparing the differences between students and employees within age groups.

## 2 Methods

This study used data from a large cohort conducted between April 2021 and May 2022. The sample comprised 600 participants from all dental schools in Canada—University of British Columbia, University of Alberta, University of Saskatchewan, University of Manitoba, University of Toronto, Western University, Université de Montréal, Université Laval, McGill University, and Dalhousie University—including students, faculty, and support staff, recruited via electronic invitations. We used a convenience sample to enroll participants from multiple dental schools aimed at ensuring a diverse and representative cohort of oral health professionals across Canada, recruiting 600 individuals at baseline who completed the initial questionnaire. The response rate at the first follow‐up was 83.5%, and by the final follow‐up (follow‐up 11), it had declined to 55.6%. This study was approved by McGill University (IRB Review Number: A12‐M69‐20B / (20‐12‐047)). Subsequently, ethical approval was obtained from the other nine universities in the study.

This study began in April 2021 with the administration of the baseline questionnaire. The timeline was shaped by the requirements of government funding and the need to navigate ethical and institutional approval processes, which determined the start date. While this period coincided with advanced COVID‐19 vaccination in Canada due to an increase in vaccine supply, the study's objectives were focused on assessing long‐term vaccination uptake and related behaviors among dental professionals.

Participants accessed a password‐protected study database and completed a questionnaire after signing a consent form. Data collection was unified across all 10 schools over ~6 weeks, with 11 follow‐ups. Eligible individuals included current trainees (dental students, dental hygienist students, dental residents, and graduate students), part‐time and full‐time academic staff (clinical and non‐clinical professors), and support staff (dental assistants, dental hygienists, sterilization technicians, receptionists, office, and administrative staff) who worked or studied at the dental school during the 11 follow‐up months.

Data were collected online through ‘LimeSurvey’. Participants completed a baseline questionnaire based on a standardized core data set established by the COVID‐19 Immunity Task Force (CITF) [22, 23]. This core set, which is a requirement for studies funded by the CITF, includes a series of pretested questions designed for consistency across studies. To align with our specific study objectives, additional relevant questions were incorporated into the follow‐up questionnaires. The adapted questionnaire was pretested in two other cohort studies examining COVID‐19 incidence among Canadian dental professionals [5, 6], ensuring its reliability and validity for our research context. The baseline questionnaire includes questions on demographics, health status, work information, and exposure potential. Monthly follow‐ups gathered information on COVID‐19 tests and symptoms, work, social activities, in‐person dental care episodes, interactions with co‐workers, vaccinations, and COVID‐19 anxiety.

## 3 Data Analysis

Data analyses included variables collected at baseline and during follow‐ups. Baseline variables were age, sex, province, role in the dental school, presence or absence of chronic conditions, including obesity, cancer, diabetes, HIV/other immune deficiency, asthma (requiring medication), chronic lung disease (non‐asthma), chronic liver disease, chronic blood disorder, chronic kidney disease, chronic neurological impairment/disease, organ or bone marrow replacement, heart condition, high blood pressure; influenza vaccination history, COVID‐19 vaccination status, vaccine doses, and vaccination dates. These vaccination‐related variables were also collected during the follow‐ups.

Descriptive statistics included frequencies, percentages, and means to describe the demographic distribution of respondents. The mean, median, standard deviation, and quartiles were calculated to analyze vaccination time distribution by age groups and role, identifying trends, spotting outliers, and patterns of vaccination delay.

We also tracked cumulative vaccination proportions and doses over time. To assess the predictors of vaccine acceptance and vaccination time, univariate logistic regression was performed to identify variables associated with vaccine acceptance and vaccination time, providing crude odds ratios (OR) and 95% confidence intervals (CI). In a subsequent step, we performed a forced‐entry multivariate logistic regression procedure providing an adjusted odds ratio (aOR) to identify covariates associated with acceptance of the COVID‐19 vaccine and vaccination time. Analyses were performed using RStudio, version 2023.06.0 + 421.

## 4 Results

### 4.1 Basic Demographic Information

The baseline survey was completed by 600 participants in April and May 2021. The median age of participants was 36 years old. Most of the participants identified as female (*n* = 411, 70%), with 52.5% (*n* = 315) reporting their primary role in dental schools as students and 47.5% (*n* = 285) as employees. By the end of May 2021, out of the 600 participants, 545 (91%) self‐reported being vaccinated with at least one COVID‐19 vaccine, and 38 (6%) did not report any vaccination. Most participants came from Quebec (*n* = 114, 19%), followed by Ontario (*n* = 103, 17%). Among the 600 respondents, 459 (76.5%) reported having no chronic conditions, and 350 (58%) reported being vaccinated for the flu vaccine in the past season (2020).

Table 1 summarizes the sociodemographic data. The data were collected from April 2021 to May 2022. During the study period, 266 participants (44.3%) were lost to follow‐up.

**TABLE 1:** Social demographic characteristics of students and employees from Canadian dental schools at baseline (*n* = 600).

Variable	*n*	%
Age group		
18–29	293	49
30–39	88	15
40–49	75	12.5
50–59	94	16
60–87	39	6.5
Missing	11	1
Sex		
Female	411	68.5
Male	179	30
Missing	10	1.5
Province of the university		
Quebec	114	19
Ontario	103	17
Others^a^	383	64
Role in the university		
Student	315	52.5
Employee	285	47.5
Presence of chronic condition^b^		
Yes	132	22
No	459	76.5
Missing	9	1.5
Flu vaccine in the past season		
Yes	350	58
No	177	29.5
Missing	73	12.5
COVID‐19 vaccine		
Yes	545	91
No	38	6
Missing	17	3
Vaccine type		
Pfizer	407	68
Moderna	52	9
AstraZeneca	24	4
Missing	117	19.5
In person dental care		
Yes	297	49.5
No	220	36.7
Missing	83	13.8

^a^ Other provinces: NS, QC, ON, MB, SK, AB and B.

^b^ Presence or not of chronic conditions—Obesity, Cancer, Diabetes, HIV/other immune deficiency, Asthma (requiring medication), Chronic lung disease (non‐asthma), Chronic liver disease, Chronic blood disorder, Chronic kidney disease, Chronic neurological impairment/disease, Organ or bone marrow replacement, Heart condition, High blood pressure.

### 4.2 COVID‐19 Vaccination in Canadian Dental Schools

The large majority of individuals in our sample accepted the COVID‐19 vaccine. Vaccination started in Canada on December 14, 2020; therefore, when our study began in April 2021, the vaccination program in Canada was advanced.

Our study revealed high vaccination acceptance among both students and employees, with a slight difference in vaccination rates between the two groups. Students comprised 52.5% (*n* = 315) of the total study sample, while employees accounted for 47.5% (*n* = 285). Among the student sample, 91.5% (*n* = 288) reported receiving at least one dose of the COVID‐19 vaccine, compared to 90% (*n* = 257) of employees.

The proportion of participants who reported being vaccinated with the COVID‐19 vaccine at study baseline was 91% (*n* = 545), with 73% (*n* = 473) reporting having received one dose and 18% (*n* = 108) two or more doses. The vaccination uptake subsequently underwent some changes, with a decrease over time in participants vaccinated with only one dose and a considerable increase in those who got the second, increasing from 18% at study baseline to up to 94% with two doses at the end of the study. The proportion of unvaccinated respondents decreased over time, with only 2.1% indicating vaccine hesitancy at the end of the study. Vaccination trends over time can be seen in Table 2.

**TABLE 2:** Vaccination trends among study participants over time.

Vaccine doses	April 2021 (*n* = 600)	October 2021 (*n* = 408)	April 2022 (*n* = 334)
One	437 (73%)	13 (3%)	7 (2%)
Two or more	108 (18%)	381 (93.4%)	316 (94.6%)
Missing	55 (9%)	17 (4%)	13 (4%)

**FIGURE 1:**
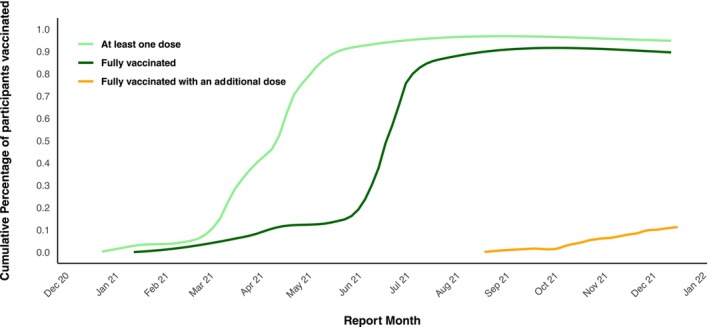
Cumulative percentage of COVID‐19 vaccination among the study population. [Color figure can be viewed at wileyonlinelibrary.com]

According to the Health Agency Canada's classification, participants who have received two doses are considered fully vaccinated. The cumulative percentage of individuals in our sample vaccinated with at least one dose and those fully vaccinated exhibited comparable patterns, with a considerable spike between March and July 2021, when most of them got vaccinated, as seen in Figure 1. Participants aged 50 to 59 represent the group with the highest vaccination coverage, but vaccine uptake was similar by sex and role.

There was no statistically significant relationship between COVID‐19 vaccine acceptance and factors such as self‐reported chronic conditions, past flu vaccination status, age groups, sex, primary role at university, and provinces. We found that individuals aged 50–59 were less likely to delay the vaccine than most sample participants (OR: 0.36, 95% CI: 0.13–0.8 and aOR: 0.16, 95% CI: 0.05–0.51, univariable and multivariable analysis respectively)—Table 3.

**TABLE 3:** Predictors of time to receive the COVID‐19 vaccine in Canadian dental universities (univariable and multivariable regression analysis results).

	Time to vaccination					
	Univariable		Multivariable			
Variables	OR	95% CI	*p*	aOR	95% CI	*p*
Sex (*n* = 534)						
Female	1					
Male	0.95	0.56–1.58	0.9	1.37	0.76–2.41	0.3
Age‐group (*n* = 534)						
18–29 y.o.	1					
30–39 y.o.	1.16	0.59–2.18	0.7	0.86	0.38–1.86	0.7
40–49 y.o.	1.65	0.86–3.08	0.12	1	0.40–2.43	> 0.9
50–59 y.o.	0.36	0.13–0.81	0.024	0.16	0.05–0.51	0.003
60–87 y.o.						
Role in the university (*n* = 538)						
Employee	1					
Student	1.21	0.76–1.95	0.4	0.81	0.39–1.72	0.6
Province (*n* = 538)						
Ontario	1					
Others^a^	3.5	1.49‐10.3	0.001	4.77	1.93–14.5	0.002
Quebec	5.81	2.29–17.9	< 0.001	7.55	2.88–23.9	< 0.001
Presence of chronic condition (*n* = 535)						
No	1					
Yes	0.96	0.53–1.65	0.9	1.35	0.68–2.57	0.4
Flu vaccine in the past season (*n* = 479)						
No	1					
Yes	0.65	0.40–1.08	0.093	0.59	0.35–1.02	0.058

Abbreviations: aOR, adjusted odds ratio; CI, Confidence Interval; OR, odds ratio; y.o., year old.

^a^ Other provinces: NS, QC, ON, MB, SK, AB and BC.

### 4.3 Vaccination Time—Since December 2020

The mean time participants took to get vaccinated for the first dose was 110 days, ~3 months (range: 1–285 days; SD: 33.5 days), while for the second dose it was 175 days, ~6 months (range: 30–364 days SD: 40.1 days), since 14 December 2020 when the vaccine was available in Canada. The median time was 113 days for the first dose (25th and 75th percentiles were 89 and 128 days, respectively) and 184 days for the second dose (25th and 75th percentile were 172 and 195 days, respectively).

**FIGURE 2:**
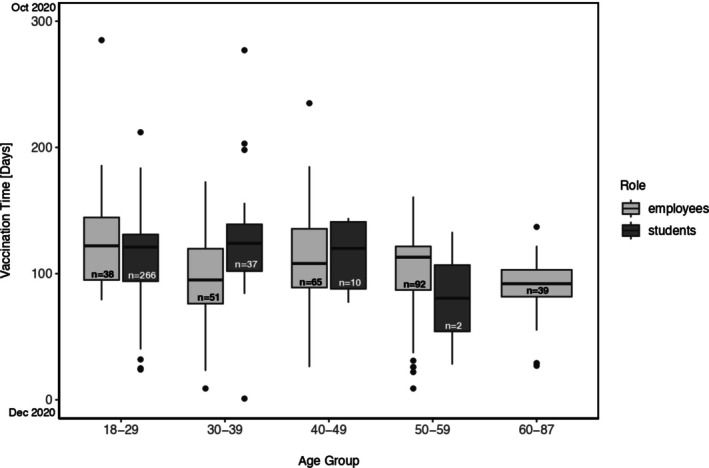
Time to vaccination with at least one dose by age group and role—since December 2020.

The Interquartile Range (IQR) of vaccination time varies across age groups and roles, indicating different levels of spread in vaccination times within each group. Several observations that deviated from the norm vaccination time were detected. These outliers represent participants who received their vaccination significantly earlier or later than the majority in their age group. Employees generally showed lower median vaccination times than students, indicating earlier vaccination. Employees also revealed more consistency in vaccination time across most age groups, with a narrower spread in vaccination times, suggesting more coherent vaccination practice. Students exhibit a broader spread in vaccination times across most age groups, suggesting more variability in when they got vaccinated. They also presented more outliers, notably for individuals in younger age groups (18–29 and 30–39 years old) who had their vaccinations later, suggesting more cases of individuals delaying their immunization. The results of the vaccination time distribution among study participants based on age group are displayed in Figure 2.

### 4.4 Lost of Follow‐Up

Our study experienced a high rate of loss to follow‐up, with up to 40% of participants dropping out. To assess potential biases, we performed a Chi‐square test to compare the characteristics of participants at baseline and at the final follow‐up. Notable differences included an increase in the proportion of employees and a corresponding decrease in the proportion of students. Additionally, there were changes in participant distribution by province, with a decline in representation from Ontario and increases from Quebec and other provinces.

## 5 Discussion

This study sheds light on vaccination uptake during the COVID‐19 pandemic among students, faculty, and support staff in Canadian dental schools, exploring vaccination trends over time. As of April 2021 (study baseline), our findings demonstrated high COVID‐19 vaccination acceptance by dental students and employees working in Canadian dental schools, with < 3% of respondents not reporting any vaccination by the end of the study (May 2022) and up to 96% of participants vaccinated. Due to very low vaccination hesitancy rates, our study did not have the statistical power to examine factors associated with hesitancy. Our findings also indicated that students were delaying the vaccine more than employees.

This is the first study, including all dental schools in Canada, that looked at actual COVID‐19 vaccination rates. One of the few studies we found in Canada on vaccination acceptance among healthcare professionals, conducted in 2021, comprised a range of HCWs, although no oral healthcare practitioners. The study revealed high vaccination acceptance among participants and low hesitancy, aligning with our findings [24]. The other two existing studies on COVID‐19 vaccination among Canadian healthcare workers, in addition to not including dental professionals, focused solely on vaccine intentions and did not provide data on vaccination coverage [25, 26]. Most studies published worldwide analyzed intention to receive the COVID‐19 vaccine instead of actual vaccination uptake [13, 14, 16, 17, 27, 28]. Our research obtained a more comprehensive overview of COVID‐19 vaccination uptake by including students and employees from different dental schools in Canada.

Our study's findings revealed high vaccination acceptance, corroborating with previous research on Quebecers' intentions to receive the COVID‐19 vaccine. Specifically, a cross‐sectional survey conducted among the Quebec population between April and December 2020 indicated a high intention to accept the vaccine, with acceptance rates ranging from 66% to 76%. The authors also revealed that the intention to be vaccinated increased with age and level of education. Our study indeed reassured these findings, aligning the intention to be vaccinated with actual vaccine acceptance [29].

In contrast to the high acceptance of the COVID‐19 vaccine from our study sample, dental students elsewhere have been documented to be hesitant for different reasons [14, 30, 31]. These findings may differ from ours due to factors such as the date when studies were conducted, the difference in sample size, sociodemographic characteristics, contextual factors, and how factors associated with vaccine hesitancy are addressed in different countries and work settings.

Our findings demonstrated no significant statistical relationship between COVID‐19 vaccine acceptance and sociodemographic or other variables. Other studies with dental students showed that gender, age, and being a dental student did not influence the willingness to receive the vaccine [14, 28]. Rock [6] also found no statistically significant relationship between COVID‐19 vaccine acceptance and age, sex, ethnicity, province of practice, or community served among Canadian dental Hygienists [6]. The fact that we did not find an association between any factors and vaccine acceptance is likely related to the high vaccination uptake in our sample and low vaccine hesitancy. Consequently, we do not have enough power to encounter such an association.

Our study outlined that participants within the group aged 50–59 years were less likely to delay the vaccination. Similar results were found in a study with Palestinian dental students, in which experience with the influenza vaccine predicted willingness to get the COVID‐19 vaccine [28]. Older age was included in Canada's priority group for vaccination [8]. It might explain why those aged 50–59 in our sample were less likely to delay the vaccine since they were included in one of the first groups eligible for vaccination.

Regarding vaccination time, students demonstrated a significant difference from the average time compared to employees, implying delays in vaccination. This could be due to various factors, like accessibility, awareness, or hesitancy. Regardless, most of our sample was vaccinated with at least one dose as of April 2021, which could be those with priority due to health conditions or being in frontline roles.

Vaccination was not mandatory in Canada but was strongly recommended by provincial and university authorities. To increase vaccination rates, local and national public health authorities implemented proof‐of‐vaccination requirements or certificates, aiming to reduce viral transmission and incentivize vaccination. By September 2021, 10 Canadian provinces (Quebec, British Columbia, Manitoba, Ontario, Nova Scotia, Alberta, New Brunswick, Saskatchewan, Newfoundland, and Prince Edward Island) had established vaccine programs. The timeline for implementing these mandates varied across provinces [10].

As of June 2021, individuals aged 12 and older were eligible for vaccination in Canada [23]. By this time, more than half of Canadians had received at least one vaccine dose, with rates progressively increasing [23]. Our sample, which included healthcare workers, likely benefited from early vaccine availability due to their priority group status. Furthermore, vaccine access and availability were fairly consistent across provinces, minimizing the likelihood of regional disparities affecting vaccination uptake in our study population.

In our study, up to 36% of participants reported not providing in‐person dental care during the study period, which may have influenced their vaccination decisions and behaviors. Those without patient contact might have perceived a lower risk of exposure, potentially impacting their motivation to get vaccinated. COVID‐19 infection rates within our study population were low. As such, prior infection likely had minimal impact on vaccine eligibility or timing. Additionally, at the time of vaccine rollout, there were no national policies prioritizing individuals based on prior infection status.

### 5.1 Strengths and Limitations

To the best of the authors' knowledge, this is the first study including all dental schools across Canada, exploring the COVID‐19 vaccination experience, coverage, and related factors. This study offers valuable insights regarding the COVID‐19 vaccination experience in several educational settings and a diverse sample. This study is part of a larger cohort study conducted during the pandemic, in which the primary goal was to document the COVID‐19 infection rates and related factors. It might introduce bias, since it was not designed to assess the vaccination experience of respondents. One limitation of our study is the declining response rate over time, which fell to 55.6% at the final follow‐up. This attrition could introduce response bias, as participants who were vaccinated may have been more likely to complete the survey, while those who were not vaccinated may have been less likely to respond. As a result, the high vaccination rates observed in our study may be overestimated. Our analysis indicated differences between those who remained in the study and those who were lost. These differences suggest bias, which may affect the generalizability of our findings. Future studies should aim to minimize loss to follow‐up and consider strategies such as weighting or sensitivity analyses to account for potential biases introduced by attrition.

## 6 Conclusion

The proportion of respondents who accepted vaccination in our study corroborates with the results from other studies performed in Canada [23, 24, 31]. Based on our findings, dental students, faculty, and support staff from Canadian dental schools demonstrated significant vaccination acceptance and low vaccine hesitancy despite the presence of some outliers among younger students indicating delays. A positive attitude toward vaccines among future dental professionals is vital for safeguarding public health and promoting a culture of responsibility, professionalism, and ethical practice within the dental community. Therefore, vaccination education should be included in their educational curriculum to prepare the workforce better.

## Data Availability

The data that support the findings of this study are available on request from the corresponding author. The data are not publicly available due to privacy or ethical restrictions.
